# Supplementary material for: On the temperature dependence of the density of states of liquids at low energies
Source: Sci Rep. 2024 Aug 13;14:18805. doi: 10.1038/s41598-024-69504-2 (PMC11322638; doi:10.1038/s41598-024-69504-2)
Supplement: Supplementary file 1 — Supplementary Information. [file 41598_2024_69504_MOESM1_ESM.pdf]

Supplementary information

On the temperature dependence of the density of states of liquids at low energies

Sha Jin, Xue Fan, Caleb Stamper, Richard A. Mole, Yuanxi Yu, Liang Hong, Dehong Yu, Matteo Baggioli
Corresponding authors: dyu@ansto.gov.au, b.matteo@sjtu.edu.cn

More details on the experimental data

In this Section, we present more details about the experimental data, the fitting procedure and the various tests performed to confirm the different low-energy scaling of the DOS in the solid and liquid phases.

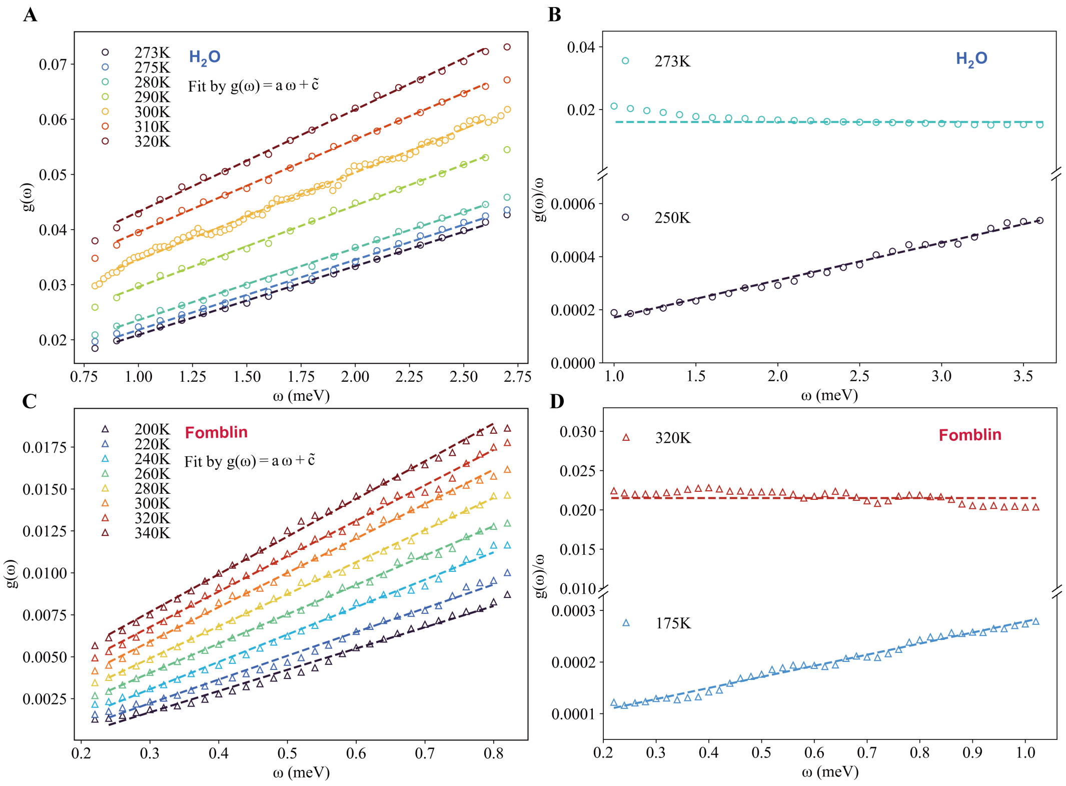

Supplementary Figure 1. **(A)** The linear fit of the experimental DOS of liquid water ($T \geq 273$ K). The dashed lines indicate the result of the fits. **(B)** The reduced form of the normalized experimental DOS of liquid water at 250 K and 273 K. The dashed lines guide the eyes towards the plateau in the liquid phase and linear behavior in the solid phase. The plateau in the data at 273 K confirms the linear scaling, $g(\omega) \propto \omega$, in the liquid phase studied in panel A. **(C)** The linear fit of the area normalized experimental DOS of Fomblin oil. **(D)** The reduced form of the experimental DOS of Fomblin oil at 175 K and 320 K. The plateau in the high-temperature data confirms the linear scaling of the DOS, $g(\omega) \propto \omega$.

In Supplementary Fig.1A we present the linear fit of the experimental DOS of water in the liquid phase. A linear function provides a good fit in the whole interval between ≈ 0.8 meV and ≈ 2.6 meV, confirming the results presented in the main text. For the linear fit of the low-frequency experimental DOS of liquid water ($T \geq 273$ K), the normalization by the area of the curves could lead to uncontrollable results due to the large spectral weight above the experimental data cutoff, 140 meV (see Fig.1A in the main text). In order to normalize the DOS properly, we resorted to a different method and used the value of the DOS at zero frequency, which, in the Brownian motion approximation, is theoretically given by the self-diffusion constant D,

$$g(0) = \frac{2mD}{k_B T}. \tag{1}$$

Since the exact zero frequency value of the DOS is not experimentally accessible, first, we fitted the low-frequency DOS of liquid water using

$$g(\omega) = a(T)\,\omega + \tilde{c}(T), \tag{2}$$

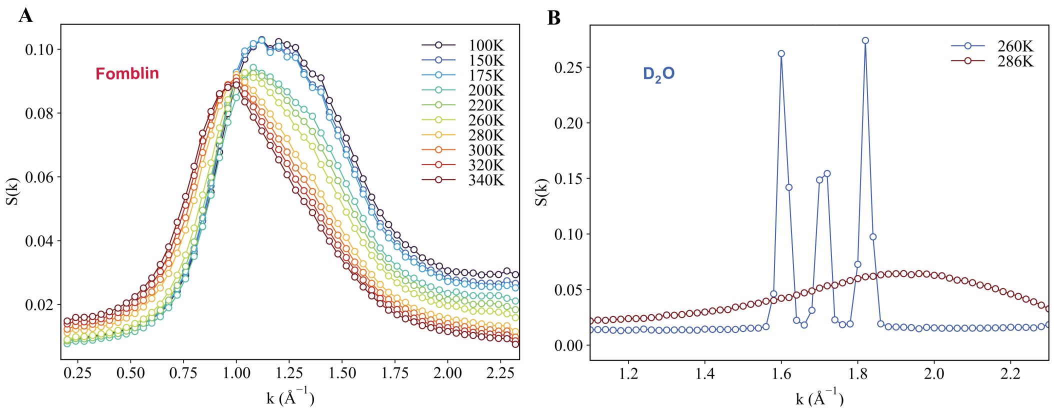

Supplementary Figure 2. The experimental structure factor $S(k)$ of Fomblin oil **(A)** and D_2O **(B)**.

and extracted the constant term $\tilde{c}(T)$. By multiplying the experimental DOS with the ratio $c(T)/\tilde{c}(T)$, where $c(T)$ is given in terms of the self-diffusion constant D as in Eq.(1), we have normalized all the curves by their value at zero frequency. The self-diffusion coefficient is obtained from the MD simulation, as presented in the Methods.

In order to confirm further the two different scalings in the liquid and solid phase, in Supplementary Fig.1B we provide a representation of the experimental DOS $g(\omega)$ reduced by the frequency ω for two representative temperatures, respectively in the liquid and solid phases. In the liquid phase, at 273 K, we observe that $g(\omega)/\omega$ is roughly flat between 1 meV and 3.5 meV, confirming the linear scaling of the DOS. On the contrary, in the solid state at 250 K, the reduced DOS shows a clear linear ramp, implying a Debye quadratic scaling for the corresponding DOS $g(\omega)$.

The linear fit of the experimental DOS of Fomblin oil, used to extract the slope presented in the main text, is plotted in Supplementary Fig.1C. The DOS curve is normalized by the total area under the curve. We notice that for the experimental data below 260 K, the linear function does not fit well the data anymore as a clear bending of the experimental data emerges. This is consistent with the analysis presented in the main text and it signals a deviation of the power-law scaling $b(T)$ from 1 as reported in Fig.2D. This deviation becomes larger by decreasing the temperature.

To further assess the existence of the two different scalings in the experimental data for Fomblin oil, we resort to the same reduced representation as done for water. In Supplementary Fig.1(D) we show the experimental data for Fomblin divided by the frequency ω at 320 K deep in the liquid phase and at 175 K, deep in the solid state. The change of behavior is evident and confirms the analysis presented in the main text.

Verification of the crystalline, liquid and glassy phases

In Supplementary Fig.2, we show the measured structure factor of Fomblin and water respectively. For Fomblin, the change of structure factors in a wide temperature range is shown in Supplementary Fig.2A. For water, the structure factor of D_2O is presented, as H_2O does not give clear diffraction peaks due to the dominating incoherent neutron scattering cross sections. Supplementary Fig.2B shows a clear first order phase transition from a crystallized structure at 260 K, with well defined sharp peaks in the structure factor, to a liquid state at 286 K, with a broad peak in the structure factor. In contrast, the structure factor of Fomblin does not exhibit any sharp peak for all the temperatures considered, but displays a similar broad peak shifting toward lower k with increasing temperature. The continuous behavior of the structure factor of Fomblin, upon moving to the low-temperature liquid state, verifies that Fomblin is not a is not a crystalline solid but it rather exhibits a short-range glassy structure with high viscosity at low temperature. This is consistent with the drastically different behavior of the low-frequency power-law of the DOS reported for the two systems in Fig.2 in the main text.

Comparison of INM data with previous literature

For completeness, we compare our results for the INM DOS of water with the other existing data in the literature. In Supplementary Fig.6, we directly compare our data (Fig.4A) with those obtained using TIP4P/2005 rigid water potential at 300K from Fig.3 in Ref. [1] and those obtained using a flexible SPC water potential at 298K from Fig.1 in Ref. [2].

This comparison reveals that the low-frequency regime is independent of the potential used and the same linear scaling $g(\omega) = a(T)\omega$ is observed. On the contrary, the DOS at higher energy depends on the potential used. Our data are very close to those obtained for flexible SPC water potential but they present marked differences with the data obtained using TIP4P/2005 rigid water potential. In particular, the DOS obtained with the rigid potential displays a much smaller number of unstable modes and a sharper peak around 65 meV.

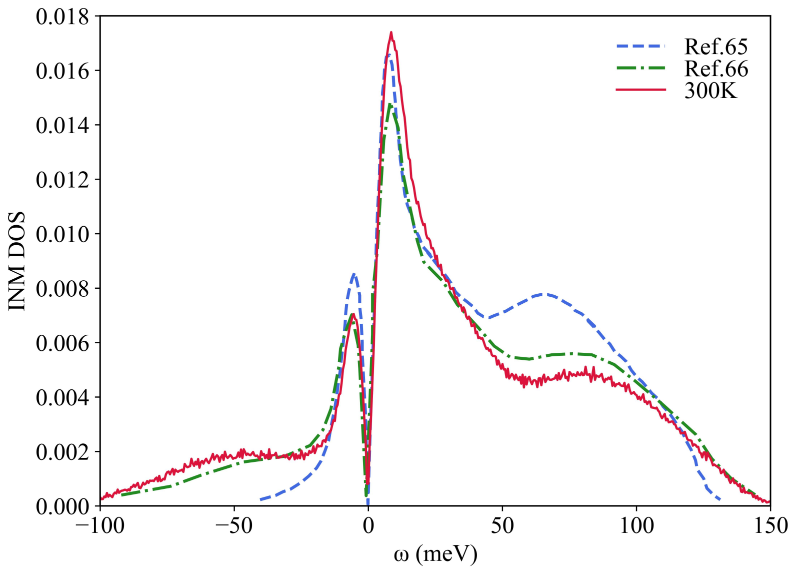

Supplementary Figure 3. The comparison of our INM DOS with the existing data in the literature. The blue and dashed curve is the INM DOS of TIP4P/2005 rigid liquid water at 300K from Fig.3 in Ref. [1]. The green dash-dotted curve is the INM DOS of flexible SPC liquid water at 298K from Fig.1 in Ref. [2]. The red solid curve corresponds to the data at 300K in Fig.4A. All the data are normalized by the area.

The linear scaling of the INM DOS

The INM spectrum of water at different temperature is shown in Fig.4A in the main text. As mentioned in the discussion of such figure, both the stable and unstable branches of the spectrum show a distinct linear scaling at low-frequency. Moreover, the linear coefficient, denoted as $a(T)$ in the main text, that governs such a scaling is the same for the two branches. In other words, the INM DOS at low frequency is symmetric under the transformation $\omega \to -\omega$.

We notice that this is a direct consequence of the definition of the INM DOS. The INM DOS is obtained from

$$g(\omega) = 2|\omega|\rho(\lambda = \omega^2), \tag{3}$$

where $\rho(\lambda)$ is the eigenvalue distribution of the instantaneous Hessian matrix. In liquids, $\rho(0) = \rho_0 \neq 0$ and therefore:

$$g(\omega) = 2|\omega|\rho_0 + \dots \tag{4}$$

where $a(T) \equiv 2\rho_0$. The function in Eq.(4) is evidently symmetric under the transformation $\omega \to -\omega$.

In order to confirm this directly, we provide a zoom of the INM spectrum around the origin $\omega = 0$ in Supplementary Fig.4 for the data at 340 K. The dashed black lines guide the eyes to the symmetric function $g(\omega) = a(T)|\omega|$ and they provide a very good fit to the simulation data. In other words, the simulation data confirm to very good accuracy that near the origin $\omega = 0$ the INM spectrum is indeed symmetric, as expected from simple theoretical arguments. We emphasize that moving away from the origin this is not anymore true, since the stable and unstable branches will become notably different at larger frequencies.

Experimental DOS versus INM DOS

In Supplementary Fig.5, we show the comparison of the experimental DOS for water and the DOS of stable INM at different temperatures. The comparisons at 280 K, 290 K, 300 K and 320 K are shown respectively in Supplementary Fig.5A, Supplementary Fig.5B, Supplementary Fig.5C and Supplementary Fig.5D. The two DOS curves have been normalized to the first peak. The two curves are quite similar in the low frequency region. As explained before, there is a difference between the two DOS, as the INM DOS does not contain the diffusion component while the experimental DOS shows a finite value at zero frequency. As expected, the two curves are closer to each other in the low frequency region for smaller temperatures. This is simply because $g(0)$, which is not captured by the normal mode analysis, diminishes with decreasing the temperature. Above approximately 20 meV, the two curves differ and the INM DOS shows much flatter and weaker bands, representing the stretching modes and librational motion. For all the temperatures considered, the slope of the linear low frequency regime is approximately the same in both DOS curves.

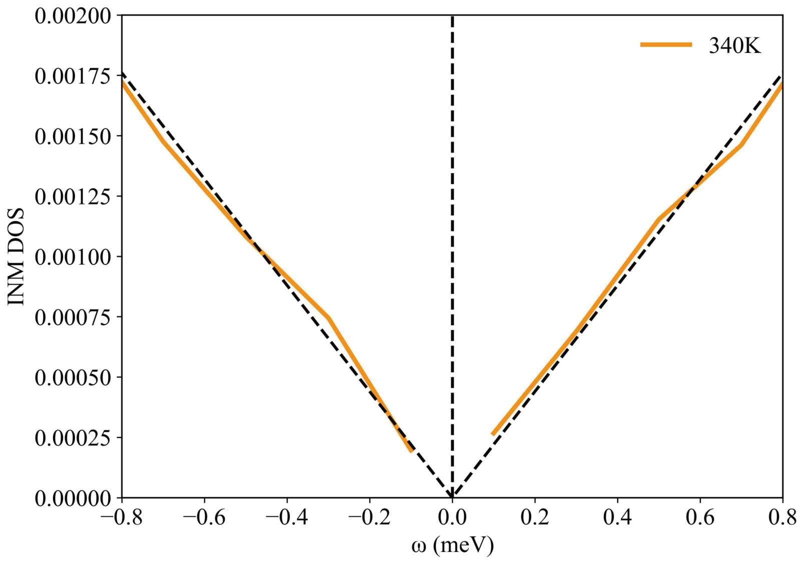

Supplementary Figure 4. A zoom of the INM spectrum of water at $T = 340$ K. In color, the simulation data. The black dashed lines guide the eyes towards the symmetric linear function $g(\omega) = a(T)|\omega|$. Similar results can be obtained at different temperatures.

Comparison with the density of state function

In this manuscript, we have focused our attention on the normal mode density of states obtained by diagonalizing the instantaneous Hessian matrix. As shown in the main text, the INM spectrum is able to capture two important features of the experimental spectrum, namely the linear scaling of the DOS at low-frequency and the temperature dependence of the corresponding coefficient. Nevertheless, as already known in the literature, the INM spectrum displays also important differences with the experimental one, that deserve further investigation. These differences are caused by the inability of the INM analysis in capturing the inherent anharmonicities of the liquid state, that are not negligible in the late-time dynamics.

A possible different approach relies on computing the Fourier transform of the velocity auto-correlation function $Z(t)$, that is usually known as the "density of state function", $\Xi(\omega)$:

$$Z(t) = \frac{\langle \vec{v}(t)\vec{v}(0)\rangle}{\langle \vec{v}(0)\vec{v}(0)\rangle}, \qquad \Xi(\omega) = \int_{-\infty}^{\infty} e^{-i\omega t} Z(t)dt. \tag{5}$$

It is known, see for example [3], that $\Xi(\omega)$ and the INM spectrum $g(\omega)$ are remarkably different below a critical frequency. This is just a reflection of the fact that, below that frequency scale, anharmonicities cannot be neglected [4] and the harmonic approximation is not justified anymore.

A complete study of $\Xi(\omega)$ and its comparison to the experimental data and the INM spectrum goes beyond the scope of this paper. Nevertheless, we have computed $\Xi(\omega)$ for water for two characteristic temperatures, 290 K and 330 K. The results are shown in Supplementary Fig.6 where the experimental data and the INM spectrum at the same temperatures are also presented. For presentation purposes, we have arbitrarily normalized all curves using the height of the first peak. We notice that a similar comparison for heavy water at 280 K has been presented in [5]. We are not aware of further results comparing these three quentities.

Let us first focus on the low-frequency regime, below the energy scale of the first peak. At low temperatures (290 K), the three curves ($\Xi(\omega)$, $g(\omega)$ and the experimental data) are rather close to each other. They all show a linear in frequency regime that extends approximately up to ≈ 5 meV. Nevertheless, $\Xi(0)$ is finite while $g(\omega)$ vanishes at zero frequency. This difference is nevertheless not that large since $\Xi(0)$ is given by the self-diffusion constant that is small at low temperature. Interestingly, $\Xi(\omega)$ shows also a small quadratic curvature at very low frequency that does not seem to be reproduced by the experimental data, and it is obviously not captured by $g(\omega)$ that is linear all the way down to $\omega = 0$.

Going to higher temperature, 330 K, $\Xi(0)$ grows as expected and the behavior of $\Xi(\omega)$ is consistently different from the experimental data below the first peak. Moreover, we can also observe that the difference between $\Xi(\omega)$ and $g(\omega)$ at low frequency is now quite large. This could be understood with the fact that anharmonicities become more relevant by increasing the temperature.

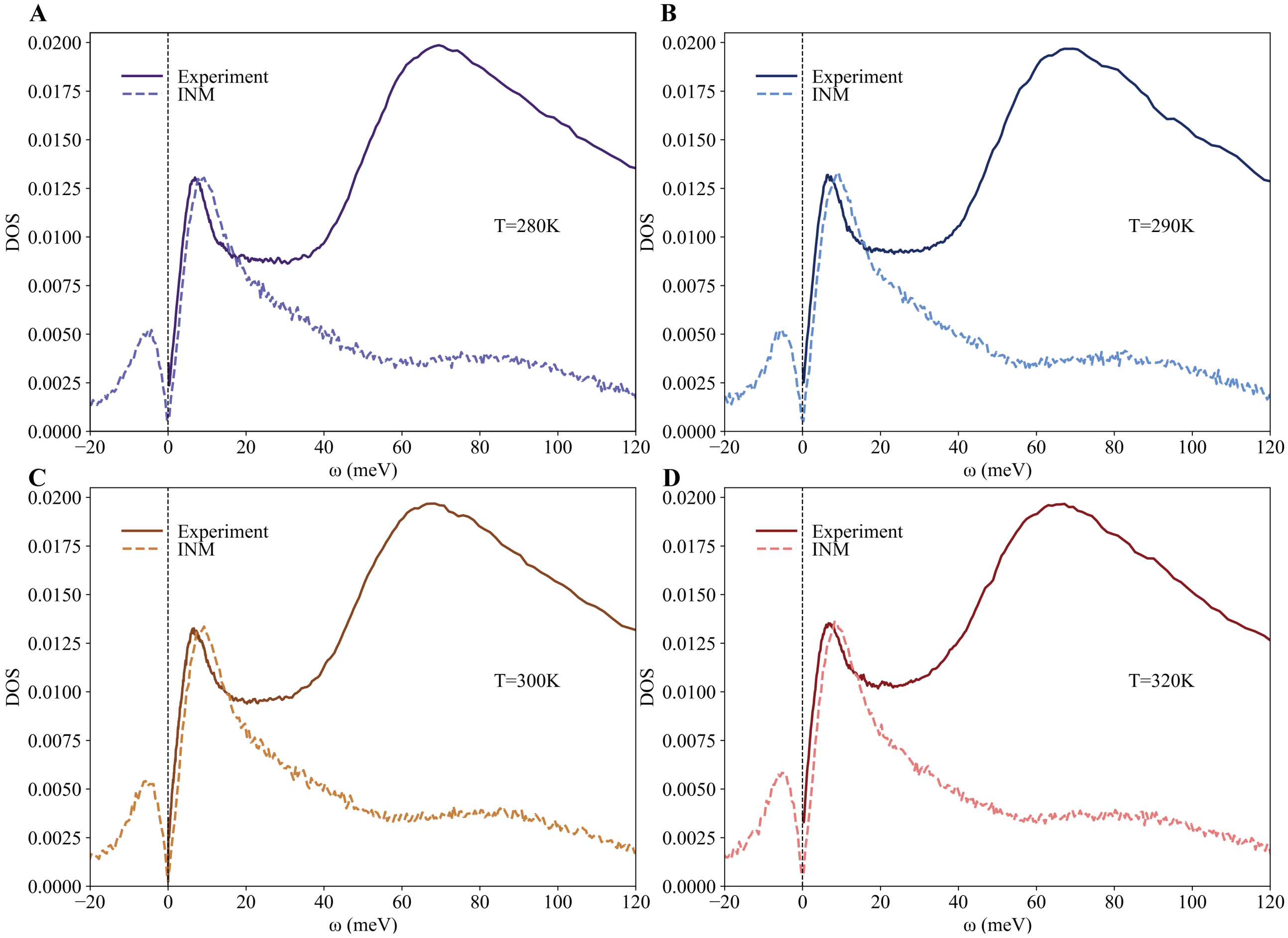

Supplementary Figure 5. The comparison between experimental DOS and INM DOS at **(A)** 280K (purple), **(B)** 290K (blue), **(C)** 300K (orange), **(D)** 320K (red).

Finally, we notice that $\Xi(\omega)$ seems to do a much better job in reproducing the experimental data at larger frequencies while the INM spectrum decays much faster and misses important features such as an evident peak around 70 mev.

We plan to perform a much more extensive comparison between $\Xi(\omega)$ and $g(\omega)$ for various systems and different temperatures in the near future.

References

1. Kuo, Y.-W., Wang, C.-W., Tang, P.-H. & Wu, T.-M. Layer structure and intermolecular vibrations of water confined within graphite nanoslits. *Chem. Phys. Lett.* **825**, 140612 (2023).

2. Huang, B.-C. & Chang, C.-H. Localization-delocalization transition of the instantaneous normal modes of liquid water. *Phys. Rev. E* **88**, 042116 (2013).

3. Seeley, G., Keyes, T. & Madan, B. From the density of states to the velocity correlation function in liquids. *The J. Phys. Chem.* **96**, 4074–4076, DOI: 10.1021/j100189a029 (1992).

4. David, E. F. & Stratt, R. M. The anharmonic features of the short-time dynamics of fluids: The time evolution and mixing of instantaneous normal modes. *The J. Chem. Phys.* **109**, 1375–1390, DOI: 10.1063/1.476690 (1998).

5. Dawidowski, J., Bermejo, F., Cabrillo, C. & Bennington, S. Generalized frequency spectra of water at both sides of the freezing transition. *Chem. Phys.* **258**, 247–255, DOI: https://doi.org/10.1016/S0301-0104(00)00128-2 (2000).

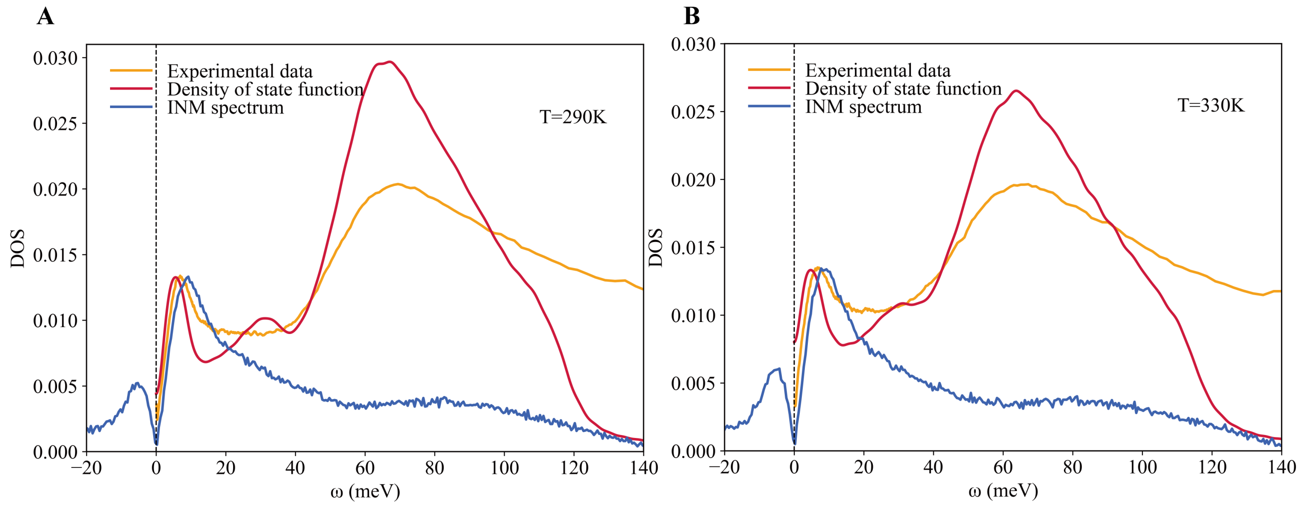

Supplementary Figure 6. The comparison between the experimental data (orange), the density of state function $\Xi(\omega)$ (red) and the INM spectrum $g(\omega)$ (blue) for water at 290 K **(A)** and 330 K **(B)**.
